# Family-Supported mHealth Coaching for Middle-Aged Men With Chronic Liver Disease and Metabolic Risk Factors: Protocol for a Pilot Randomized Controlled Trial

**DOI:** 10.2196/95490

**Published:** 2026-10-30

**Authors:** Seoeun Bae, Seojin Lee, Yeonsoo Jang

**Affiliations:** 1 College of Nursing and Brain Korea 21 FOUR Project Yonsei University Seoul Republic of Korea; 2 Mo-Im Kim Nursing Research Institute, Institution for Innovation in Digital Healthcare College of Nursing Yonsei University Seoul Republic of Korea

**Keywords:** family relations, feasibility studies, liver diseases, mobile apps, randomized controlled trial, self-management

## Abstract

**Background:**

Chronic liver disease (CLD) remains a substantial public health burden, particularly among middle-aged men with metabolic risk factors. Sustained lifestyle modification is essential for preventing disease progression, but long-term self-management in this population is often suboptimal. Although mobile health (mHealth) interventions may support self-management, most are patient-centered and rarely incorporate structured family participation.

**Objective:**

This study aims to develop a family-supported mHealth coaching intervention for middle-aged men with CLD and metabolic risk factors, evaluate the feasibility of the intervention and trial procedures, and explore preliminary app usability and changes in patient- and family-related outcomes through a pilot randomized controlled trial (RCT).

**Methods:**

This 2-arm, parallel-group pilot RCT was conducted at the Liver Center of Severance Hospital, Yonsei University Health System, Seoul, Republic of Korea. Eligible patients were male outpatients aged 40 to 64 years who had CLD for at least 6 months and at least 1 metabolic risk factor. The planned sample size was 82 patients, with 41 patients per group. Enrolled patients were randomly assigned in a 1:1 ratio to a 12-week family-supported mHealth coaching intervention or usual outpatient care plus a standardized educational video delivered via text message. Guided by the information-motivation-behavioral skills (IMB) model, the intervention integrates app-based self-monitoring, individualized feedback, online coaching, and opportunities for family participation. The feasibility and acceptability of the intervention and trial procedures will be evaluated. Primary outcomes are self-management behavior, perceived social support, depressive symptoms, and sleep quality; secondary outcomes include physical and clinical indicators and family experiences. Outcomes were assessed at baseline (week 0) and postintervention (week 12). Between-group changes will be examined using appropriate parametric or nonparametric tests, with an analysis of covariance used for baseline-adjusted comparisons. Effect estimates will be reported with 95% CIs and interpreted as preliminary. Complete-case analyses will be conducted without imputation of missing data.

**Results:**

This study was funded in March 2023 and received institutional review board approval on April 9, 2025 (approval 4-2025-0117). The first participant was enrolled in May 2025, and data collection was completed in March 2026. A total of 52 patient participants were enrolled and randomized, of whom 35 completed the 12-week follow-up, corresponding to an overall attrition rate of 32.7%. Data analysis and preparation of the main trial manuscript are currently underway, with submission for publication anticipated in winter 2026.

**Conclusions:**

This pilot RCT evaluates an IMB model–informed, family-supported mHealth coaching intervention integrating app-based self-monitoring and individualized coaching. The findings will provide preliminary evidence regarding feasibility, acceptability, and changes in patient- and family-related outcomes and will inform intervention refinement and the design of a future definitive RCT.

**Trial Registration:**

Clinical Research Information Service KCT0010494; https://tinyurl.com/75m6c7c6

**International Registered Report Identifier (IRRID):**

DERR1-10.2196/95490

## Introduction

### Background

Chronic liver disease (CLD) is characterized by progressive deterioration of liver function persisting for more than 6 months and may result from alcohol consumption, viral infections, autoimmune disorders, or metabolic diseases [1]. According to an analysis of Global Burden of Disease 2019 data, digestive diseases continue to impose a substantial global health burden [2]. In South Korea, liver disease remains a leading cause of mortality, particularly among middle-aged men [3-6].

Metabolic risk factors, including obesity and type 2 diabetes mellitus (DM), are key contributors to the progression of metabolic dysfunction-associated steatotic liver disease [7]. These factors are associated with both liver disease progression and increased cardiovascular risk [8,9]. In contrast, lifestyle modifications—such as weight control, dietary management, and regular physical activity—have been shown to reduce the risk of liver fibrosis and slow disease progression [10-12]. These findings underscore the importance of self-management among patients with CLD and concomitant metabolic risk factors.

Self-management refers to the cognitive, behavioral, and emotional capacity required to manage the physical, psychological, and social consequences of chronic illness in daily life [13]. However, men with liver disease often demonstrate poorer self-management and a higher risk of fibrosis progression than women [14,15]. Occupational demands and constraints related to social roles may further impede self-management among middle-aged men [14]. Therefore, sustained self-management support that reflects individuals’ everyday life contexts is essential for effective liver disease management.

Family functioning and social support have been consistently associated with improved self-management adherence and better health outcomes in patients with CLD [16-18]. Greater family support is associated with higher self-efficacy, better self-management, and improved quality of life [19,20]. These findings suggest that self-management may be strengthened more effectively through family participation than through individual efforts alone.

Mobile health (mHealth) technologies have increasingly been used to support treatment adherence and self-management among patients with chronic diseases. A systematic review has reported improved adherence outcomes and generally high usability and feasibility while also noting potential barriers related to digital access and literacy [21,22]. App-based interventions have been shown to improve disease management and reduce hospital readmissions [23-25]. However, most existing interventions remain primarily patient-centered, with limited integration of family involvement [26,27], despite evidence that family participation can facilitate self-management by providing ongoing support and reinforcing behavioral changes in chronic illness care [28]. Previous studies have also reported several patient-perceived barriers to mHealth adoption, including usability issues, privacy concerns, technological limitations, and gaps in digital literacy [29]. A family-supported mHealth approach may therefore offer a promising strategy for strengthening self-management among individuals with CLD. However, the feasibility and acceptability of this approach have not been established among middle-aged men with CLD and metabolic risk factors or among their family members. A pilot evaluation is therefore needed to refine the intervention and trial procedures before conducting a definitive randomized controlled trial (RCT).

### Objectives

This pilot study aimed (1) to develop a 12-week mHealth coaching intervention designed to incorporate family support for middle-aged men with CLD and metabolic risk factors and (2) to evaluate the feasibility of the intervention and associated trial procedures in terms of recruitment, retention, intervention adherence, and data completeness. Preliminary app usability and changes in patient- and family-related outcomes will also be explored to inform a future definitive RCT.

## Methods

### Study Design

This study was designed as a 2-arm, parallel-group pilot RCT with 1:1 allocation at the patient-family dyad level. The primary focus was the feasibility of the intervention and trial procedures, with patient- and family-related outcomes assessed exploratorily. This protocol follows the SPIRIT (Standard Protocol Items: Recommendations for Interventional Trials) 2025 guidelines [30]; the completed checklist is provided in Multimedia Appendix 1.

### Participants and Recruitment

Middle-aged men with CLD and metabolic risk factors who were attending the Liver Center of Yonsei University Hospital in Seoul, Republic of Korea, and 1 family member per patient were recruited. During routine visits, attending physicians introduced the study to potentially eligible patients and referred those who expressed interest to trained research staff. With the patients’ agreement, the research staff reviewed relevant information in the electronic medical record (EMR) to confirm basic eligibility. Eligible patients and their family members who voluntarily agreed to participate provided written informed consent before enrollment as a dyad. Textbox 1 presents the inclusion and exclusion criteria for both groups.

**Eligibility criteria for patients and family members.**

**Inclusion criteria**

**Patients**

- Men aged 40 to 64 years
- Diagnosis of chronic liver disease for at least 6 months, including hepatitis B virus infection, hepatitis C virus infection, metabolic dysfunction-associated steatotic liver disease (MASLD), or liver cirrhosis
- At least 1 metabolic risk factor as defined in the EASL-EASD-EASO Clinical Practice Guidelines for the management of MASLD [31]. Metabolic risk factors included the following: hypertriglyceridemia (triglycerides ≥150 mg/dL) or low high-density lipoprotein cholesterol (<40 mg/dL); hypertension (systolic blood pressure ≥130 mm Hg or diastolic blood pressure ≥85 mm Hg); impaired fasting glucose (fasting plasma glucose ≥100 mg/dL); abdominal obesity (waist circumference ≥90 cm); obesity (BMI ≥25 kg/m^2^); or current use of medications for any of these conditions [32,33]
- Ability to use a smartphone app and complete online questionnaires

**Family members**

- Adults aged ≥19 years
- Ability to use a smartphone app and complete online questionnaires

**Exclusion criteria**

**Patients**

- Decompensated liver cirrhosis
- Diagnosis of hepatocellular carcinoma or other malignancy within the past 5 years and ongoing active treatment (eg, chemotherapy, radiotherapy, or immunotherapy)

### Randomization, Allocation, and Blinding

Patient-family dyads were randomly assigned in a 1:1 ratio to the intervention or control group using block randomization with a block size of 4. The allocation sequence was generated in advance using a web-based randomization system [34] by a designated researcher who was not involved in participant recruitment or enrollment. The prespecified allocation sequence was applied to each dyad in the order in which written informed consent was obtained. Group allocation was concealed from the research staff responsible for participant recruitment and consent.

Because of the nature of the coaching app intervention, participants and intervention providers could not be blinded to group assignment. To minimize the potential influence of group allocation on baseline assessment, group assignment was disclosed by an intervention provider who was not involved in participant recruitment or enrollment to patients and family members only after the patient had completed the baseline survey. The analyst conducting the outcome analyses was not involved in participant recruitment, group allocation, or data collection and will not be blinded to group assignment during the analyses. To minimize potential analytical bias, the analyses will be conducted according to the prespecified statistical analysis methods described in this protocol.

### Sample Size

Sample size was calculated using G*Power (version 3.1.9.7) [35], with self-management behavior as the primary outcome. A medium effect size (*f*=0.25) [36] was assumed based on findings from a previous RCT that reported Cohen *d* values of 0.45 to 0.59 [25]. For a repeated-measures ANOVA with a group-by-time interaction, 2 groups, and 2 measurement points, α=.05 and 80% power, the minimum required sample size was estimated to be 68 patients (34 per group). Allowing for an anticipated 20% attrition rate, the planned sample size was increased to 82 patients (41 per group). Under the original design, 1 family member per patient was also expected to participate. Although the sample size was based on an efficacy-oriented calculation, clinical outcomes in this pilot RCT will be interpreted as exploratory findings rather than as definitive evidence of intervention effectiveness.

### Intervention Development

#### Theoretical Framework

The intervention was informed by relevant self-management and behavior change interventions for chronic diseases, including liver disease. Core components identified from the literature—health education, self-monitoring, goal-setting, and individualized feedback—were organized according to the information-motivation-behavioral skills (IMB) model [37].

The information component provided structured education on liver disease, potential complications, nutrition, physical activity, and lifestyle modification. Personal motivation was promoted through individualized goal-setting, coaching, and feedback, while social motivation was supported through structured family participation. The behavioral skills component focused on self-monitoring and practical skills for managing diet, physical activity, medication adherence, sleep, alcohol consumption, and smoking, as applicable. Patients established individualized behavioral goals with their coach, and family members were encouraged to support goal attainment. Dietary and physical activity goals were tailored to each patient’s needs and circumstances.

#### Smartphone App Development

The smartphone app was developed to deliver the 12-week family-supported mHealth intervention and support participant self-management. The app provided a digital environment for implementing the intervention (Figure 1). Patients recorded self-management behaviors, including dietary intake, physical activity, medication adherence, sleep, alcohol consumption, and smoking, as applicable. Family members independently recorded observations of patients’ behaviors using dedicated input fields. Both patients and family members could review accumulated records to support self-management and goal-setting.

**Figure 1:**
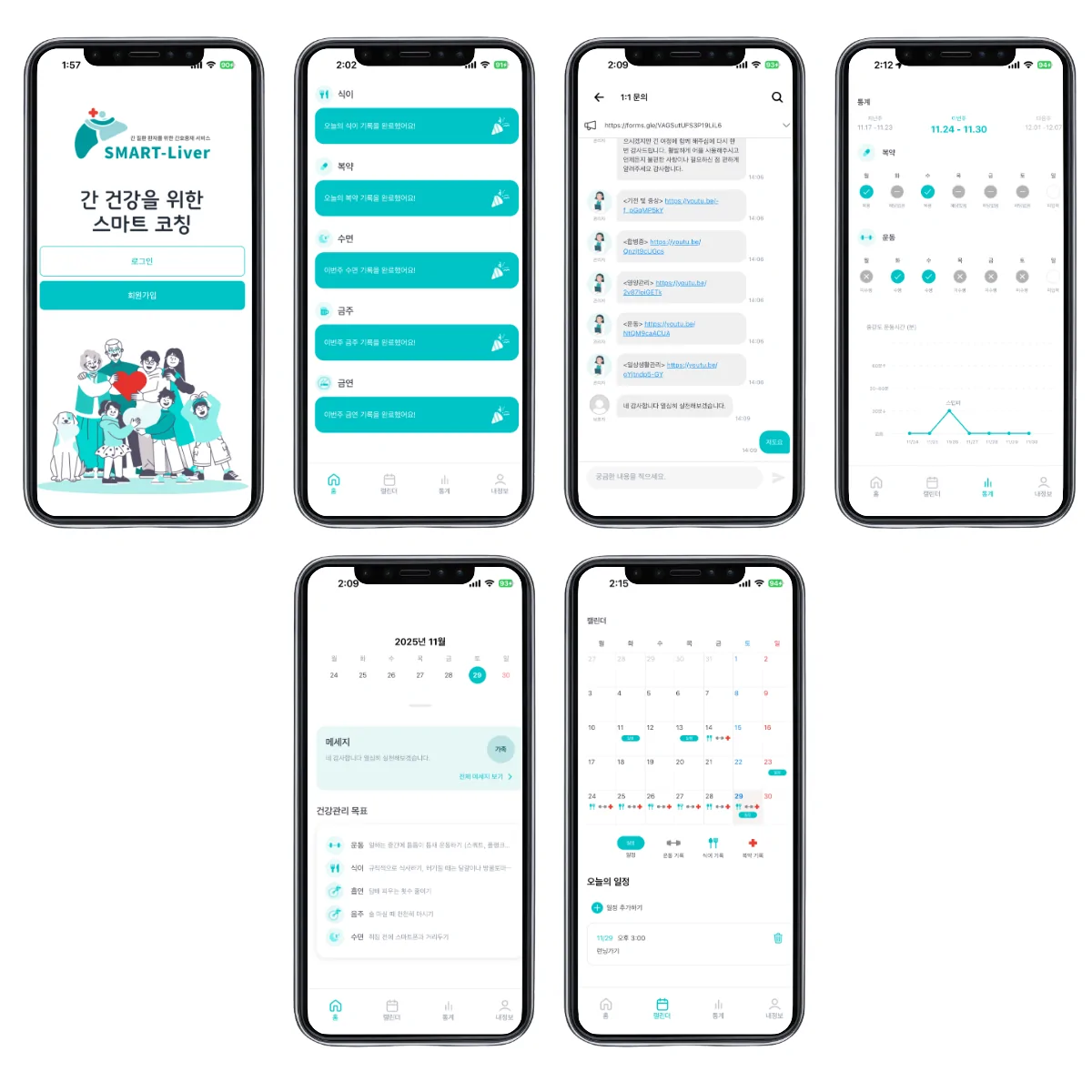
User interface and key functions of the family-supported mobile health app.

The app used a client-server architecture comprising iOS and Android mobile apps for participants, a central server for data storage and exchange, and a web-based administrative interface for the research team. Researchers used the administrative interface to monitor engagement and behavioral changes and provide individualized feedback and coaching. Direct messaging enabled 2-way communication, allowing participants to ask questions about the intervention and self-management and receive responses from the research team.

#### Usability Evaluation

Usability was assessed before and after the RCT intervention to identify problems and inform app refinement using the System Usability Scale (SUS) [38]. The initial target was 5 participants, based on Nielsen’s formative-testing recommendation [39].

Convenience sampling was used to recruit individuals meeting the RCT eligibility criteria (Textbox 1). Of the 5 initially invited participants, 4 completed the evaluation: 1 installed the app via a text-message link and completed testing before implementation of the intervention, while 3 downloaded the app from their operating system’s app store after the app was released and completed testing after the 12-week intervention period. Respondents comprised 3 male patients and 1 female family member, aged 41-58 years; 1 used an iOS device, and 3 used Android devices. Participants received instructions for app installation, account creation, and use. All assessments were conducted following actual use on personal smartphones; no devices were supplied, and no static images were used.

The mean SUS score was 86.88 (SD 8.75). An open-ended online survey identified difficulties with sedentary-time recording, requests for more varied content and engagement features, and perceived benefits of dietary self-monitoring. Before trial implementation, researchers reviewed app functions, content, data-entry procedures, and access controls and addressed identified technical and usability issues. Postintervention feedback informed plans for further refinement.

#### Expert Content Validity Assessment

An expert panel of 7 health care professionals evaluated the content validity of the app-based intervention components and materials. The panel comprised 2 gastroenterologists experienced in liver disease care and education and 5 registered nurses with at least 5 years of gastroenterology experience. The panel evaluated the relevance of app content and items across 4 domains—self-monitoring, coaching, feedback, and health information—using a 4-point Likert scale ranging from 1=not relevant to 4=highly relevant. The item-level Content Validity Index (I-CVI) was calculated as the proportion of experts rating an item as either 3 or 4 [40]. All evaluated items had an I-CVI of 1.00, indicating unanimous agreement regarding their relevance. Based on the experts’ feedback, several items were revised to improve clarity, including the addition of descriptions and examples to distinguish vigorous-intensity activity, moderate-intensity activity, and muscle-strengthening exercise. The intervention content was finalized following these revisions.

### Procedure

Throughout the study, participants in both groups continued their usual clinical care and prescribed treatments. Any changes in medical treatment were made at the discretion of their treating physicians.

#### Intervention Group

The intervention was delivered over 12 weeks as a family-supported, dyadic self-management program. Patients and their family members jointly participated in structured goal-setting, self-monitoring, and online video coaching throughout the intervention period. Coaching sessions were scheduled at weeks 0, 4, 8, and 12 to establish, review, and refine individualized self-management goals. The overall intervention flow is presented in Figure 2.

To reduce the risk of contamination, recruitment and informed consent procedures were conducted separately for each patient-family dyad in a private room. After completion of the baseline assessment, each dyad was privately notified of its group allocation by text message. Intervention group participants were then provided with instructions on app installation, account creation, and use of its main features. Once the app had been installed and an individual account had been created, access to the app was granted. The research team then scheduled an initial online coaching session with each dyad. During the 30-minute Zoom (Zoom Video Communications) session, the patient and family member established individualized self-management goals for the first 4 weeks based on the patient’s baseline assessment. Within 3 days after the initial session, a basic educational video addressing liver disease mechanisms, potential complications, lifestyle management, physical activity, and dietary management was delivered through the app’s messaging feature. No group-based sessions were conducted; all coaching and app-based components were delivered separately to each patient-family dyad.

Following the baseline coaching session, intervention group participants began using the intervention-specific components, including app-based self-monitoring, individualized coaching and feedback, structured family participation, and 2-way communication with the research team. Patients recorded physical activity, dietary intake, and medication adherence daily and sleep, alcohol consumption, and smoking weekly, as applicable. Family members independently recorded their observations of the patients’ self-management behaviors and supported the patients in working toward their individualized goals.

**Figure 2:**
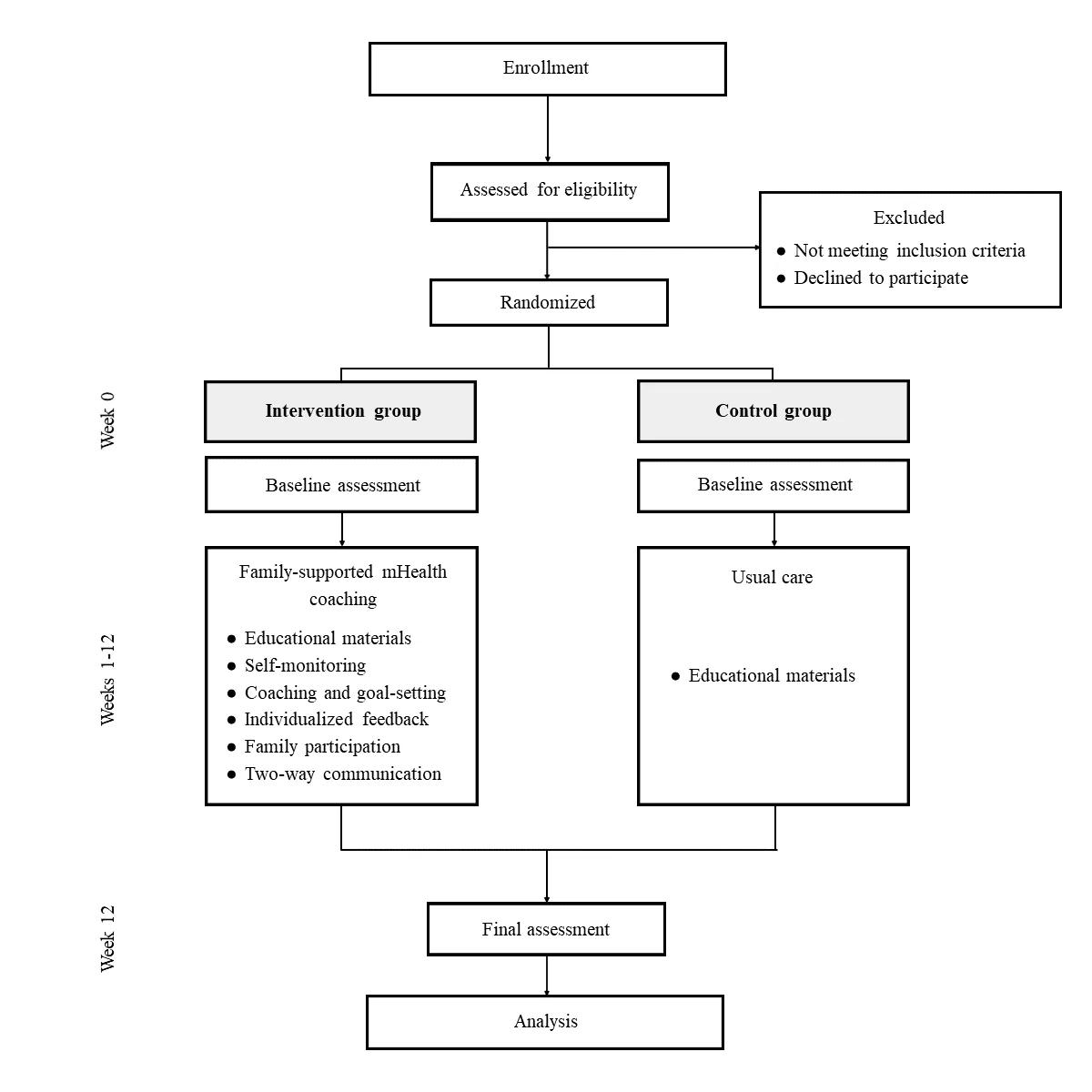
Flowchart of the intervention protocol. mHealth: mobile health.

#### Intervention Fidelity

A standardized intervention manual was developed to promote consistent delivery throughout the 12-week intervention period. The manual specified the activities to be completed at each time point, the required content of each coaching session, and the procedures for monitoring participant progress. All coaching sessions were delivered by a single trained intervention provider, who followed the manual to minimize variability in intervention delivery across dyads.

Completion of scheduled coaching sessions and required intervention components was monitored throughout the intervention period. The research team held weekly meetings to review intervention progress, identify omitted or inconsistently delivered components, and discuss deviations from the intervention protocol. Corrective actions were implemented when necessary to improve adherence to the standardized procedures.

#### Control Group

Participants in the control group received usual outpatient care for liver disease, including specialist consultations and clinical monitoring determined by their treating physicians. After completion of the baseline assessment, each dyad was privately notified of its allocation by text message and received a link to the same basic educational video provided to the intervention group.

Patients completed brief self-report surveys on alcohol consumption, smoking, and sleep through text-message links at weeks 4, 8, and 12. In addition to the brief week 12 survey, patients and family members completed the full postintervention assessment of prespecified patient- and family-related outcomes. These assessments were for data collection only and included no individualized feedback or coaching. Participants in the control group received no login credentials and had no access to the app or its intervention-specific features.

### Data Collection and Management

#### Outcome Measures

Prespecified primary outcomes were self-management behavior, perceived social support, depressive symptoms, and sleep quality; secondary outcomes comprised patients’ physical and clinical indicators and participating family members’ family experience. Outcomes were assessed at baseline (week 0) and postintervention (week 12). Table 1 presents the schedule of enrollment, interventions, and assessments in accordance with SPIRIT 2025 [30].

**Table 1:** Schedule of enrollment, interventions, and assessments.

Trial period				Screening		Baseline (week 0)		Week 4		Week 8		Postintervention (week 12)
**Enrollment**												
	Eligibility screening			✓								
	Informed consent			✓								
	Randomization					✓						
**Intervention group**												
	App installation and educational materials					✓						
	Coaching session (video)					✓		✓		✓		✓
	Self-monitoring via app^a^					✓		✓		✓		✓
	Weekly surveys^b^					✓		✓		✓		✓
**Control group**												
	Educational materials					✓						
	Monthly surveys^b^					✓		✓		✓		✓
**Assessments**												
	Demographics					✓						
	**Primary outcomes**											
		Self-management			✓						✓	
		Perceived social support			✓						✓	
		Depression symptoms			✓						✓	
		Sleep quality			✓						✓	
	**Secondary outcomes**											
		Physical and clinical indicators^c^			✓						✓	
		Family experience			✓						✓	

^a^Diet, physical activity, and medication.

^b^Sleep, alcohol, and smoking.

^c^Body composition and laboratory parameters.

#### Data Collection Procedures

Study data were obtained from EMRs, self-reported questionnaires, and app-generated records. Physical and clinical indicators were extracted from patient EMRs in both groups at the scheduled assessments. When measurements were unavailable at the corresponding outpatient visit, the closest available measurements were used in accordance with the study protocol. Patient- and family-reported outcomes were collected through an online survey platform. Baseline clinical characteristics included liver disease duration, etiology, current medication use, Child-Pugh scores, and liver scan parameters. Details of the study measures, assessment instruments, and assessment populations are presented in Table 2.

**Table 2:** Study measures, assessment instruments, and assessment population.

Study measure				Assessment instruments or measures	Assessment population
Demographics				Age, marital status, education level, employment status, and monthly household income	Patients
Demographics				Age, sex, marital status, relationship to the patient, cohabitation status, education level, employment status, shift work status, monthly household income, and previous liver disease–related health education	Family members
Baseline clinical and lifestyle				Alcohol consumption, smoking status, sleep duration, perceived health status, liver disease history, comorbidities, medication and supplement use, previous liver disease–related health education, Child-Pugh score, and liver scan parameters	Patients
**Primary outcomes**					
	Self-management		Revised NAFLD-SMQ^a^		Patients
	Perceived social support		MSPSS^b^		Patients, family members
	Depression symptoms		PHQ-9^c^		Patients, family members
	Sleep quality		PSQI-K^d^		Patients
**Secondary outcomes**					
	**Physical and clinical indicators**				
		Body composition measures	BFP^e^, SMM^f^, BMI, PhA^g^		Patients
		Anthropometric measures	Height, BW^h^, WC^i^		Patients
		Vital signs	BP^j^		Patients
		Laboratory parameters	Total cholesterol, HDL-C^k^, LDL-C^l^, triglycerides, ALP^m^, AST^n^, ALT^o^, total protein, albumin, GGT^p^, total bilirubin (also FPG^q^ and HbA_1c_^r^ as applicable)		Patients
	**Family-related outcomes**				
		Family experience	FIES:CI^s^		Family members

^a^NAFLD-SMQ: Nonalcoholic Fatty Liver Disease Self-Management Questionnaire.

^b^MSPSS: Multidimensional Scale of Perceived Social Support.

^c^PHQ-9: Patient Health Questionnaire-9.

^d^PSQI-K: Korean version of the Pittsburgh Sleep Quality Index.

^e^BFP: body fat percentage.

^f^SMM: skeletal muscle mass.

^g^PhA: phase angle.

^h^BW: body weight.

^i^WC: waist circumference.

^j^BP: blood pressure.

^k^HDL-C: high-density lipoprotein cholesterol.

^l^LDL-C: low-density lipoprotein cholesterol.

^m^ALP: alkaline phosphatase.

^n^AST: aspartate aminotransferase.

^o^ALT: alanine aminotransferase.

^p^GGT: γ-glutamyl transferase.

^q^FPG: fasting plasma glucose.

^r^HbA_1c_: glycated hemoglobin.

^s^FIES:CI: Family Integration Experience Scale: Chronic Illness.

During the intervention, behavioral records and brief surveys were collected through the app or text-message survey links at the intervals specified in the Intervention Group and Control Group subsections. Reminder messages supported scheduled survey completion; intervention-group participants also received reminders for coaching sessions and intervention-related activities.

#### Primary Outcomes

Self-management behavior was measured using the revised Nonalcoholic Fatty Liver Disease Self-Management Questionnaire [41]. Perceived social support was evaluated using the Korean version of the Multidimensional Scale of Perceived Social Support [42], originally developed by Zimet et al [43]. Depressive symptoms were measured using the Korean version of the Patient Health Questionnaire-9 [44], originally developed by Spitzer et al [45]. Sleep quality was evaluated using the Korean version of the Pittsburgh Sleep Quality Index [46], originally developed by Buysse et al [47].

#### Secondary Outcomes

Secondary outcomes comprised physical and clinical indicators for patients and family experience for participating family members. Physical indicators included height, body weight, BMI, waist circumference, body fat percentage, skeletal muscle mass, phase angle, and blood pressure obtained from study assessments and EMRs, as applicable. Biochemical markers extracted from EMRs included total cholesterol, high-density and low-density lipoprotein cholesterol, triglycerides, alkaline phosphatase, aspartate and alanine aminotransferases, γ-glutamyl transferase, total protein, albumin, total bilirubin, and fasting plasma glucose. Glycated hemoglobin was additionally extracted for patients with DM.

Family experience was assessed using a Korean translation of the Family Integration Experience Scale: Chronic Illness, developed by Meiers et al [48]. Family-related findings will be interpreted separately and not as evidence of reciprocal patient-family effects.

#### Data Quality Control

Trained research staff extracted clinical data from EMRs using standardized procedures and a predefined variable list. Collected data were cross-checked against relevant source data to identify discrepancies and ensure accuracy and consistency. App-entered data were stored on a secure server accessible only to authorized research personnel, with periodic system checks to identify technical issues and reduce the risk of data loss. During data collection, the principal investigator (YJ) and research team regularly reviewed data completeness, accuracy, and consistency at weekly meetings.

### Harms

This pilot trial is considered a minimal-risk behavioral intervention. Potential risks include temporary psychological discomfort, time burden, fatigue, technical difficulties, and privacy concerns. Before implementation, intervention content and procedures were reviewed using prior research and expert content validation to minimize burden and harm.

Participants can report concerns during coaching sessions, follow-up assessments, or direct communication with the research team. The principal investigator and research team will review each concern and take appropriate action, including technical support, further assessment, or intervention interruption, modification, or discontinuation. Outcome assessments may continue after intervention discontinuation unless the participant requests withdrawal or safety considerations require otherwise. No formal adverse-event grading or causality-classification system is prespecified. Serious or medically significant events will be promptly reported to the principal investigator and institutional review board in accordance with institutional requirements. Participant-reported concerns and unintended effects will be summarized descriptively to inform intervention feasibility and acceptability.

### Statistical Analysis

All analyses will be performed using SPSS Statistics (version 29.0; IBM Corp). Baseline characteristics will be summarized descriptively by randomized group without statistical significance testing.

The primary exploratory analysis will compare change scores from baseline to postintervention between the intervention and control groups using independent-samples *t* tests or Mann-Whitney *U* tests, as appropriate. Between-group effect estimates will be reported with 95% CIs and effect sizes (Cohen *d* or *r*). Within-group changes will be examined as supplementary analyses using paired-samples *t* tests or Wilcoxon signed rank tests. An analysis of covariance will also be conducted as a supplementary analysis to compare postintervention outcomes between groups, adjusting for the corresponding baseline values. All statistical tests will be 2-sided with a significance level of *P*<.05. The primary analysis population will comprise participants with both baseline and postintervention data for the outcome being analyzed. Missing values will not be imputed; their extent and reported reasons will be summarized by randomized group. Recruitment, retention, intervention adherence, and data completeness will be summarized descriptively. No separate per-protocol analysis is planned.

### Ethical Considerations

This study was approved by the institutional review board of Yonsei University Health System, Severance Hospital (approval 4-2025-0117), covering the RCT, smartphone app development, usability evaluation, and expert content validation. The trial was conducted in accordance with the Declaration of Helsinki. Trained research staff obtained written informed consent from all patient and family participants before enrollment, after providing written study information and sufficient time for questions. Consent covered survey and EMR data use, including related ancillary analyses. Participation in all study activities, including the RCT involving patients and family members, the usability evaluation, and expert content validation, was voluntary. All participants could withdraw at any time without penalty or effect on their medical care or participation. Information collected through these study activities was deidentified, handled confidentially, and stored securely in accordance with institutional policies. Although not prespecified in the study protocol, periodic independent reviews were conducted to confirm that no ineligible participants had been enrolled and that informed consent documentation and study procedures were appropriately followed in accordance with the approved protocol. Participants who completed the 12-week study received compensation, and those who completed the usability evaluation received separate compensation.

### Dissemination Policy

The findings of this pilot RCT will be reported in accordance with the CONSORT-eHEALTH (Consolidated Standards of Reporting Trials of Electronic and Mobile Health Applications and Online Telehealth) checklist [49]. Results will be disseminated through publication in peer-reviewed journals and presentation at academic conferences. No individually identifiable information will be disclosed.

## Results

The study received institutional review board approval on April 9, 2025 (approval 4-2025-0117). The first participant was enrolled in May 2025, and the final follow-up assessment was completed in March 2026. A total of 52 patients were enrolled and randomized, of whom 35 completed the 12-week follow-up and 17 discontinued participation. Attrition rates were 30.8% in the intervention group and 34.6% in the control group. Reasons for discontinuation were categorized as personal circumstances, including work, scheduling, or family matters (n=7), difficulties or burden related to study procedures or participation (n=4), and loss to follow-up or nonresponse (n=6). Data analysis and preparation of the main trial manuscript are currently underway, with submission for publication anticipated in winter 2026.

## Discussion

### Anticipated Findings

This pilot RCT is expected to provide preliminary evidence regarding the feasibility of an IMB model–based mHealth coaching intervention designed to incorporate family support for middle-aged men with CLD and metabolic risk factors. The findings will help identify practical considerations related to recruitment, retention, intervention adherence, data completeness, and the delivery of family-supported digital coaching. Preliminary changes in patient- and family-related outcomes will be interpreted as exploratory and used to refine the intervention and trial procedures for a future definitive RCT.

The IMB model provides an established theoretical foundation for behavior change interventions in chronic disease management. Theory-informed interventions have supported adherence and health behaviors in DM [50,51], chronic obstructive pulmonary disease [52], and coronary artery disease [53]. In CLD, limited disease-related knowledge [54-56] and difficulty sustaining motivation for lifestyle modification [57] remain important self-management challenges. Inadequate engagement is also a persistent limitation of smartphone-based health interventions [58]. By addressing information, personal motivation, social motivation, and behavioral skills within a structured digital environment, this study may provide useful insights into the feasibility of applying the IMB model to family-supported CLD self-management.

### Strengths and Limitations

A key contribution of this study is the integration of structured family participation into an mHealth self-management intervention. The intervention was designed to extend individual-centered care by embedding social motivation and opportunities for family support within a technology-supported environment. Prior research suggests that family involvement may facilitate lifestyle modification [59], improve psychological outcomes such as reduced anxiety and greater self-efficacy [60], and support self-management in mHealth interventions [61]. Despite this growing body of evidence [62-65], family members have often been involved only peripherally, and sustaining their engagement over time remains challenging [66-68]. By incorporating family participation as a core intervention component rather than an adjunct [69,70], this pilot RCT will provide preliminary evidence on the feasibility of delivering a family-supported mHealth self-management intervention and inform strategies for strengthening family engagement in a future definitive trial.

This study has some limitations. First, the feasibility and generalizability of this mHealth-based intervention may be limited among individuals with low digital literacy or restricted access to mobile devices. Second, recruitment of patient-family dyads remained challenging. Enrollment was limited when family members lived separately in another region, were substantially older or younger than the patient, or were unable to use a mobile phone. As a result, the planned sample size was not reached. The reduced sample may have limited statistical power and the precision of effect estimates; therefore, the clinical outcomes will be interpreted as exploratory. Third, the usability evaluation involved a small number of participants assessed at different stages of app use, which may limit the generalizability of the usability findings.

### Conclusions

This protocol describes a single-center pilot RCT of an IMB model–based, family-supported mHealth coaching intervention for middle-aged men with CLD and metabolic risk factors. The intervention integrates app-based self-monitoring, individualized coaching, and family participation to support sustained self-management. Of the target enrollment of 82 patients, 52 were enrolled, and 35 completed the 12-week follow-up. Given the smaller-than-planned sample, clinical outcomes will be interpreted as exploratory rather than as definitive evidence of intervention effectiveness. The findings will inform refinement of the intervention, recruitment and retention strategies, and the design of a future adequately powered RCT. Following completion of the ongoing analysis, the findings will be disseminated through peer-reviewed publication and academic presentations.

## Appendix

Multimedia Appendix 1 SPIRIT 2025 checklist.

## Abbreviations

CLD: chronic liver disease
CONSORT-eHEALTH: Consolidated Standards of Reporting Trials of Electronic and Mobile Health Applications and Online Telehealth
DM: diabetes mellitus
EMR: electronic medical record
I-CVI: item-level Content Validity Index
IMB: information-motivation-behavioral skills
mHealth: mobile health
RCT: randomized controlled trial
SPIRIT: Standard Protocol Items: Recommendations for Interventional Trials
SUS: System Usability Scale

## Data Availability

The datasets generated or analyzed during this study will be available from the corresponding author upon reasonable request.
